# Parliament and the Profession

**Published:** 1916-12-02

**Authors:** 


### Parliament and the Profession.

### Pay of Soldiers in Hospital.

A number of questions have recently been put to the Secretary to the War Office on the subject of the pay of soldiers while in hospital; it is suggested that it often happens that when a married man is stopped 7d. a day for hospital treatment, and has made an allotment of 6d. a day, nothing is left for himself. The latest questioners were Mr. W. Thorne and Mr. Pringle, who sought information as to how the principle of assessment was carried out. Mr. Forster said that where a man has to go to hospital in consequence of wounds or disease incurred through service, there is no stoppage at all; deduction is made only when a patient's hospital treatment is necessitated through his own fault. Mr. Forster replied that discretion in all these cases lies with the patient's commanding officer.

### Use of Asylums for Wounded.

The Home Secretary gave some information as to the work done by the Board of Control in converting asylums for the reception and treatment of wounded and incapacitated soldiers. Mr. Samuel stated that thirteen asylums and a part of a fourteenth, containing over 15,000 lunatic patients, who were transferred to other asylums, had been adapted for hospital purposes. About 23,600 beds were thus provided, and up to date they had been used for 100,000 cases.

### Nurses and the Kanturk Guardians.

Mr. T. M. Healy asked the Chief Secretary for Ireland if he was consulted by the Local Government Board when the Kanturk Guardians repealed its resolution not to give posts to women who had not served as nurses at the Front. Mr. Duke said he was not consulted in the matter, and that the Local Government Board had no control over the Guardians except where given by Statute, which was not the case in the circumstances referred to.

### Shell-shock and Nerve Troubles.

Mr. Forster informed Sir E. Cornwall that a scheme was under examination for providing beneficial open-air employment on the land for officers and men sent home suffering from shell-shock and nervous trouble.

### Doctors' National Health Remuneration.

Mr. Alden asked Mr. Roberts if he was aware that practitioners were unable to draw balances due for 1915 from the London Insurance Committee unless they signed a form of receipt relinquishing their legal rights under the regulations and otherwise; a state of affairs which was causing irritation and dissatisfaction. Mr. Roberts said he was making inquiries into the precise form of receipt and discharge adopted, but that *prima facie* the giving of a receipt in full discharge did not appear to be unreasonable.

